# Zinc Ionophore (Clioquinol) Inhibition of Human ZIP1-Deficient Prostate Tumor Growth in the Mouse Ectopic Xenograft Model: A Zinc Approach for the Efficacious Treatment of Prostate Cancer

**DOI:** 10.23937/2378-3419/3/1/1037

**Published:** 2016-01-09

**Authors:** Renty B. Franklin, Jing Zou, Yao Zheng, Michael J. Naslund, Leslie C. Costello

**Affiliations:** 1 Department of Oncology and Diagnostic Sciences, Dental School, The Greenbaum Cancer Center, University of Maryland, Baltimore, Maryland, USA; 2 Department of Oncology and Diagnostic Sciences, Dental School, University of Maryland, Baltimore, Maryland, USA; 3 Department of Periodontics, Dental School, University of Maryland, Baltimore, Maryland, USA; 4 Division of Urology, University of Maryland School of Medicine, Baltimore, Maryland, USA

**Keywords:** Prostate cancer, Zinc, Ionophore, Clioquinol, Chemotherapy, ZIP1, Citrate, Tumor suppressor, Prostate malignancy

## Abstract

Prostate cancer remains the second leading cause of cancer deaths in males. This is mainly due to the absence of an available efficacious chemotherapy despite decades of research in pursuit of effective treatment approaches. A plausible target for the treatment is the established clinical relationship that the zinc levels in the malignant cells are markedly decreased compared to the normal epithelium in virtually all cases of prostate cancer, and at all stages malignancy. The decrease in zinc results from the downregulation of the functional zinc uptake transporter, ZIP1; which occurs during early development of prostate malignancy. This is an essential requirement for the development of malignancy to prevent the cytotoxic/tumor-suppressor effects of increased zinc on the premalignant and malignant cells. Thus prostate cancer is a ZIP1-deficient malignancy. This relationship provides the basis for a treatment regimen that will facilitate the uptake and accumulation of zinc into the premalignant and malignant cells.

In this report we employed a zinc ionophore (clioquinol) approach in the treatment of mice with human ZIP1-deficient prostate tumors (ectopic xenograft model). Clioquinol treatment resulted in 85%inhibition of tumor growth due to the cytotoxic effects of zinc. Coupled with additional results from earlier studies, the compelling evidence provides a plausible approach for the effective treatment of human prostate cancer; including primary site malignancy, hormone-resistant cancer, and metastasis. Additionally, this approach might be effective in preventing the development of malignancy in individuals suspected of presenting with early development of malignancy. Clinical trials are now required in leading to the potential for an efficacious zinc-treatment approach, which is urgently needed for the treatment of prostate cancer.

## Introduction

In recent years, about 230,000 new cases and about 30,000 deaths due to prostate cancer occur annually in the USA [1] which is the second leading cause of cancer deaths in males. Thus it becomes evident that the morbidity and mortality of prostate cancer constitute a major health issue. The major problem is the absence of effective treatment of advanced stage malignancy and metastasis; and for the hormone-resistant cancer following androgen-deprivation treatment. In addition, the tentative suspicion of unconfirmed early malignancy or the presence of low volume and low grade malignancy is often followed by “active surveillance”; during which no treatment is employed until the appearance of malignant progression. When malignancy is confirmed, invasive treatment regimens are employed. In all of the above conditions, no efficacious chemotherapy exists.

As we have described in many research and review articles, a marked decreased zinc in malignancy is a hallmark characteristic in virtually all cases of prostate cancer. In 1981 we first identified zinc as an inhibitor of prostate citrate metabolism [2] and in 1999 we first identified zinc as an inhibitor of prostate malignant cell proliferation [3] and subsequent studies have established the requirement of decreased zinc for the development and progression of prostate malignancy. This has been the basis for our proposal over the past ~20 years that the zinc relationship provides a plausible target for the development of a zinc treatment approach for prostate cancer. Recent reported studies from our group and other investigators have continued to verify, strengthen, and expand this zinc relationship. Rather than presenting another extensive description of the background information, we refer the reader to our most recent extensive reviews [4–6].

For this present study, the most important established relationships are: 1) the zinc levels are always markedly decreased in human prostate cancer as compared to the normal peripheral zone (where ~90% of malignancy arises); 2) the accumulation of zinc in the malignant cells exhibits cytotoxic/tumor suppressor effects; and 3) ZIP1 is the important functional zinc uptake transporter that is down regulated in the malignant cells in situ in prostate cancer; which protects the malignant cells from accumulation of cytotoxic levels of zinc. Thus, we now characterize human prostate cancer as a “ZIP1-deficient malignancy”.

This new and critical understanding must be recognized and considered in any zinc treatment approach for prostate cancer. It implies that the restoration of increased cytotoxic zinc levels in the malignant cells for treatment of prostate cancer must include a process or vehicle that facilitates the entry of zinc into the ZIP1-deficent malignant cells. To demonstrate the plausibility of this relationship in a recent preliminary study [6], we employed a zinc ionophore (clioquinol; 5-chloro-7-iodo-8-quinolinol) for the treatment of ectopic human ZIP1-deficient prostate tumor growth in nude mice. The results demonstrated effective tumor suppression by clioquinol treatment. To confirm and extend the preliminary results, we now present another study, which further establishes the concept of an ionophore approach for the zinc treatment of human ZIP1-deficient prostate cancer. Thus the consistent and compelling clinical and experimental evidence should lead to clinical trials regarding the efficacy of clioquinol or an alternative appropriate ionophore for the treatment of human prostate cancer.

## Methods

### ZIP1-deficient prostate tumor xenograft model

In order to represent the ZIP1-deficient malignancy status that exists in human prostate cancer, we established a ZIP1 knockdown PC3 cell line. PC3 cells obtained from the American Type Collection were grown and maintained in RPMI 1640 medium with L-Glutamine and HEPES supplemented with 10% Fetal Bovine Serum and 1% penicillin-Streptomycin mixture. The cells were stably transfected using 1 or 2 μg of a ZIP1 shRNA vector from Dharmacon, followed by selection of transfected cells in the presence of G418. The ZIP1 knockdown was verified by Western blot analysis of whole cell extracts (described in [6]). Transfected cells were plated in RPMI 1640 medium, grown to near confluence, collected and used to raise xenograft tumors in nude mice.

Nude mice were maintained according to the protocol approved by the University of Maryland Institutional Animal Care and Use Committee. Stably transfected PC3 cells were collected by collagenase digestion and suspended in 10% Matrigel. A suspension of 1×10^6^ cells in 100 μl of HBSS was injected subcutaneously into both flanks of five 4-week-old male nude mice. Clioquinol was dissolved in Intralipid 20% and 33 mg/Kg in 250 μl injected i.p. every other day. Five Control animals were injected with the same volume of Intralipid 20% without clioquinol beginning one week after the beginning of tumor growth in all animals. Animals were weighed every other day and tumors measured using calipers once each week for 8 weeks. The tumor volume was calculated using the formula V = l × W^2^; where l equals the length (greatest dimension) and W equal the width of tumors.

### Citrate and Zinc measurements

Citrate and zinc concentrations were determined on tumor extracts. Tumors were excised and homogenized in 7% TCA. The homogenates were centrifuged and an aliquot was collected for assay. Citrate was determined by the Furth-Hermann reaction [7] with slight modification. 20 μl of extract was added to 400 μl of acetic anhydride; and heated to 60^º^C for 10 min. The reaction was allowed to cool to room temperature and 50 μl of pyridine was added; and the reaction reheated to 60^º^C for 40 min. The reaction mix was cooled to room temperature and the samples read in a microplate reader at 425 nm.

The concentration of zinc in the extracts was determined using the fluorescent indicator Zinquin ethyl ester. Sample (100 μl) was added to 3 μl of 25 mM zinquin in ethyl alcohol. Water was added to a final volume of 200 μl and the fluorescence read at 355 nm/485 mM (excitation/emission).

### Statistical analysis

The data were subjected to t-test analyses to determine statistical significance.

## Results and Discussion

**Figure 1:**
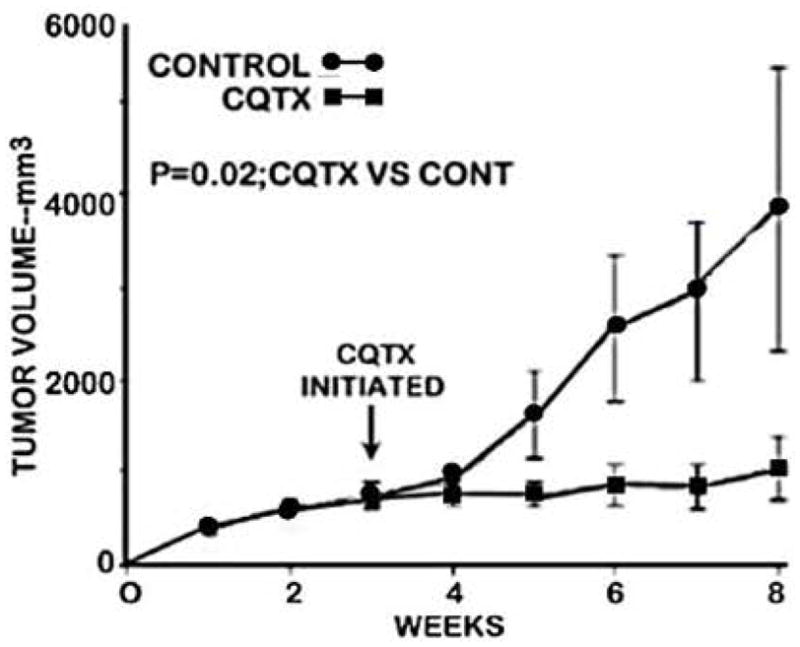
The effect of clioquinol treatment on the growth of ectopic human ZIP1-deficient prostate tumors in the mouse xenograft model.

**Figure 2:**
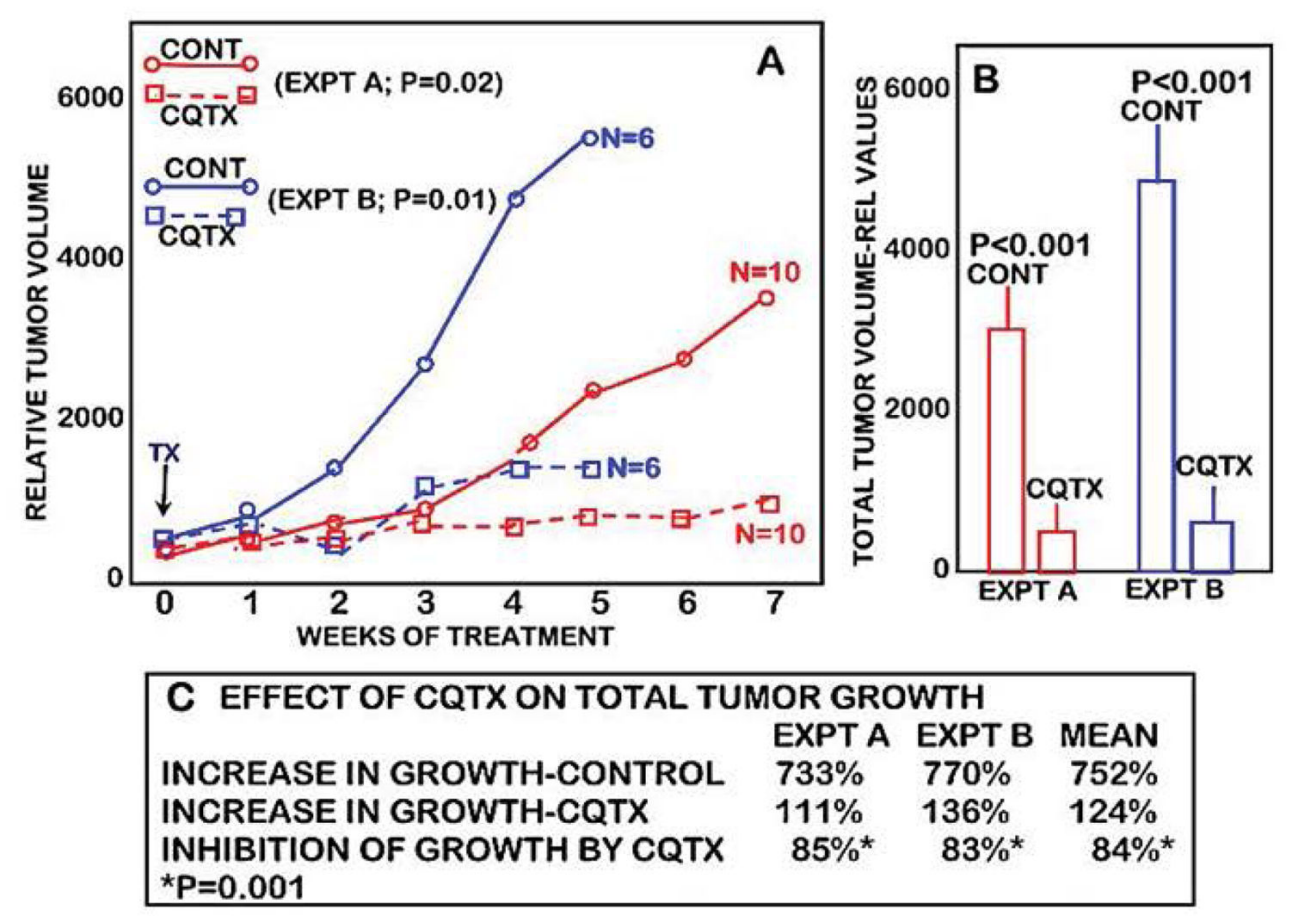
The comparative effects of two studies of the clioquinol treatment on the growth of ectopic human ZIP1-deficient prostate tumors in the mouse xenograft model. EXPT A is the current study (Figure 1); and EXPT B is the earlier Costello et al study reported in [6]. Both studies were conducted essentially under the same conditions.

Figure 1 shows the tumor growth rate of clioquinol-treated versus control (vehicle-treated) animals. It is clearly evident that clioquinol treatment markedly suppressed the growth of ZIP1-deficient tumors. Following the initiation of treatment, tumor growth in clioquinol-treated animals increased by 111% as compared to 733% increase in untreated control animals; thereby representing an 85% inhibition of tumor growth by clioquinol treatment (Figure 2B and Figure 2C). Figure 2 also shows the results of clioquinol treatment in this study (Expt A) as compared to our recent initial preliminary study (Expt B) [6]. Both studies involved the same conditions regarding the inoculation of ZIP1-deficient PC-3 cells to establish tumor development; and involved the same clioquinol treatment regimen. Figure 2A shows that both experiments exhibited essentially identical profiles of the tumor suppressor effects of clioquinol. Despite the fact that the control tumor growth rate and tumor size were greater in the earlier study, a consistent ~85% suppression of tumor growth by clioquinol treatment occurred, independent of the tumor burden.

Two related hallmark characteristics of essentially all cases of prostate cancer are the decrease in zinc and in citrate in malignancy (for reviews see [4–6]. The decrease in citrate results from the decrease in zinc; which then removes the zinc-inhibition of citrate oxidation. Therefore the determination of tumor zinc and citrate concentration is important. Table 1 shows a significant increase in citrate in tumors from CQ-treated animals compared to tumors from untreated animals.

There was also the expected increase in zinc that should accompany the increase in citrate. However the 34% increase in zinc was not statistically significant (P = 0.1). Similarly in our preceding experiment, clioquinol treatment also resulted in a 62% increase in zinc, which was not statistically significant. However the accompanying increase in citrate is evidence for a functional increase in zinc. Our studies with prostate [8] identified zinc as a specific inhibitor of m-aconitase; which is the only known physiological m-aconitase inhibitor. As such, the increase in citrate results from increased cellular/mitochondrial zinc via its inhibition of m-aconitase. Moreover such inhibition results from the increase in the cellular exchangeable reactive pool of zinc (i.e. the mobile pool); which generally represents about 5% of the total cellular zinc. This relationship requires that the effectiveness of the change in cellular zinc be demonstrated by cellular actions that are zinc–specific; as is its inhibition of m-aconitase. In addition, this clioquinol effect on increasing the tumor citrate level is evidence that clioquinol increased the cellular mobile pool of zinc.

These studies now establish the highly effective suppression of ZIP1-deficient prostate tumor growth by clioquinol treatment in the mouse ectopic xenograft model. The results also corroborate our concept that the restoration of high zinc levels in the tumor cells in vivo will exhibit cytotoxic/tumor suppressor activities. We previously described [6] that in vitro exposure of the ZIP1-deficient PC-3 cells to physiological concentrations of zinc had little effect on the cellular uptake of zinc, and thereby had no effects on cell growth. However in the presence of clioquinol, cellular uptake of zinc occurred and resulted in inhibition of cell growth.

Collectively, these observations establish that the cytotoxic/tumor suppressor effect is the result of: 1) clioquinol transport of extracellular zinc across the plasma membrane and into the Zip1-deficient tumor cells; and 2) the intracellular release of zinc for its various cellular effects; as we anticipated based on the clioquinol zinc binding affinity (formation constant) of log Kf~8 [9]. Clioquinol effects have often been attributed to cations other than zinc, which it also binds. Therefore, it is important that we provided the evidence that establishes its cytotoxic/tumor suppressor effects being mediated via its action as a zinc ionophore. This is also evident from the clioquinol effect with ZIP1-deficient prostate cells being the same as zinc treatment effects in the absence of clioquinol in ZIP1-expressing cells [6].

Although some in vitro and in vivo studies [10–12] have demonstrated clioquinol-induced cytotoxic/tumor suppresser effects on tumor cells, none has employed tumor cells that have been identified as being representative of the status of the ZIP-family zinc uptake transporter as exists in situ in the human cancer. This is an important issue since malignant prostate cell lines in culture exhibit re-expression of ZIP1, although the malignant cells in situ in human prostate cancer exhibit down-regulation of ZIP1 (for review [4–6]). This also applies to hepatocellular cancer and pancreatic cancer [13,14]. Thus, such studies have questionable translational value; and must include the conditions that are representative of the in situ status of prostate cancer.

Our studies have employed the ionophore, clioquinol, because of its properties such as the zinc binding affinity; and because it has been employed in humans with low toxicity at effective dosage [15,16]. In our studies with mice, we employed concentrations equivalent to that used in humans, without any overt indication of adverse effects. The results demonstrate that, under appropriate conditions, a zinc ionophore approach could provide a successful chemotherapy for prostate cancer.

**Figure 3:**
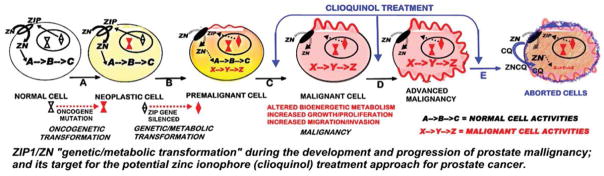
The ZIP1/Zn “genetic/metabolic transformation” during the development and progression of prostate malignancy; and its target for the potential zinc ionophore (clioquinol) treatment approach for prostate cancer. Steps A, B, C, D represent the oncogenetic development of prostate malignancy malignancy. The blue arrows represent the target sites for the clioquinol treatment that facilitates the increased uptake and accumulation of zinc resulting in the cytotoxic effects of zinc shown as step E.

In addition, the results also support our concept and proposal that a treatment regimen that facilitates the uptake and accumulation of zinc in the ZIP1-deficient malignant prostate cells will result in cytotoxic/tumor suppression effects. This also requires that, within the cell, the accumulated zinc must exist as an exchangeable reactive pool of zinc. Figure 3 represents the targeting of the development of prostate cancer for zinc treatment with the ionophore approach. It includes the understanding of prostate cancer carcinogenesis (which applies to all cancers [17]) that the oncogenetic initiation of the malignant process must include downstream “genetic/metabolic” transformations of the neoplastic cell leading to the development and progression of the premalignant and malignant cells [4–6]. The requirement for the downregulation of ZIP1 and decreased zinc accumulation in the development of prostate malignancy represents such a “genetic/metabolic” event. Since this transformation is evident in PIN and in the malignant cells, it provides an ideal target for prevention of early development of malignancy and for treatment of advanced stage malignancy; as represented in figure 2. It also becomes evident that the ionophore approach is essential to deliver the circulating plasma zinc into the ZIP1-deficient premalignant and/or malignant cells; and that within the cell, the zinc must be in the exchangeable reactive form so as to exhibit its cytotoxic/tumor-suppressor effects.

We have focused on clioquinol as representative of a zinc ionophore approach, and have now demonstrated its potential effectiveness for treatment of prostate cancer. However, these conditions might also be achieved by other agents or mechanisms that will facilitate the uptake of zinc into the ZIP1-deficient cells in exchangeable reactive forms, which could provide effective treatment of prostate cancer.

## Conclusions

Despite decades of research, an efficacious chemotherapy for advanced stage prostate cancer does not exist; nor for prevention of the early development of malignancy. Thus prostate cancer remains a leading cause of cancer deaths in males. In regard to this issue, extensive and consistent clinical and experimental evidence accumulated over the past ~ 40 years have established the zinc relationships that we have described above. Moreover, no reported substantial and corroborated clinical evidence exists that contradicts this ZIP1/zinc prostate will deliver zinc into ZIP1-deficient tumors; thereby effectively inhibiting tumor growth. This provides a plausible approach for the effective treatment of human prostate cancer; including primary site malignancy, hormone-resistant cancer, and metastasis. Additionally, this approach might be effective in preventing the development of malignancy in individuals suspected of presenting with early development of malignancy. Thus, clinical trials are now justified and required to establish the value of this zinc ionophore approach for treatment of prostate cancer. In the meantime, in the absence of an efficacious chemotherapy, thousands of males continue to develop prostate cancer and suffer from its morbidity and ultimately death.

## Figures and Tables

**Table 1:** The effect of clioquinol treatment on the tumor citrate and zinc levels

	CITRATE (nmol/mg protein)			ZINC (ng/mg tissue)		
	CONT	CQTX	INCR	CONT	CQTX	INCR
**EXPT A**	3.5 ± 0.6	7.4 ± 1.2	111%*	6.4 ± 0.4	8.6 ± 1.2	34%
**EXPT B**	----------	----------	----------	9.5 ± 0.4	15.4 ± 3.7	62%
